# Human adipose-derived stromal/stem cells demonstrate short-lived persistence after implantation in both an immunocompetent and an immunocompromised murine model

**DOI:** 10.1186/scrt532

**Published:** 2014-12-18

**Authors:** Hitesh Agrawal, Hulan Shang, Anna Parker Sattah, Ning Yang, Shayn M Peirce, Adam J Katz

**Affiliations:** Department of Pediatric Cardiology, Baylor College of Medicine, Texas Children’s Hospital, 6621 Fannin Street, Houston, TX 77030 USA; Department of Surgery, Division of Plastic and Reconstructive Surgery, University of Florida, 1600 SW Archer Road, Gainesville, FL 32610 USA; George Washington University Hospital, 2121 I St NW, Washington, DC 20052 USA; Department of Biomedical Engineering, University of Virginia, 415 Lane Road, Room 2041, Charlottesville, VA 22903 USA; Department of Plastic Surgery, University of Virginia, 1215 Lee Street, Charlottesville, VA 22903 USA

## Abstract

**Introduction:**

Mesenchymal cells are emerging as a promising cell platform for regenerative therapies. However, the fate of cells after transplantation in many different disease settings and tissue beds remains unclear.

**Methods:**

In this study, human adipose-derived stromal/stem (ASCs) cells were fluorescently labeled with a membrane dye and injected into both immunocompetent and immunocompromised mouse strains. Cells were injected either as single cell suspensions, or as self-assembling spheroids. In parallel, cells were purposefully devitalized prior to injection and then implanted in the opposite side in a randomized fashion. These ‘control’ groups were included to determine whether the fluorescent membrane dye would remain localized at the injection site despite the use of nonviable cells. Cell implants and the surrounding tissues were harvested on days 3, 10 and 21 after *in vivo* delivery and evaluated in a blinded manner. Injection sites were analyzed by fluorescent microscopy, and human cell numbers were quantified using PCR detection of a human-specific endogenous retrovirus (ERV-3). Host response was evaluated by immunofluorescent staining of macrophages.

**Results:**

ERV-3 quantification showed that 95% of the human cells that were viable when they were injected were undetectable at the three-week time-point. Although fluorescent signal persisted for the entire study period, further analysis revealed that much of this signal was located within host macrophages.

**Conclusions:**

These results suggest that human ASCs survive for less than three weeks after injection into even immunocompromised mice, and call into question the notion that human ASCs are immuno-privileged and capable of surviving for extended periods in xenogeneic and/or allogeneic models.

## Introduction

As the promise of cell-based therapies begins to transition to the clinic, a clear understanding of the survival, localization and identity of administered cells over time remains elusive but of great interest. A major limitation relates to various technical challenges associated with the reliable identification and tracking of cells *in vivo*. Even in the context of preclinical animal models, the sensitive and specific identification of human cells after implantation presents certain challenges. Although a number of methods have been described, most – if not all – should be re-evaluated in the context of recent findings related to cell-derived vesicles, or microparticles [1, 2]. These elements encompass a heterogeneous spectrum of membrane-bound structures ranging in size from 20 nm to 1 μm, released by an ever-expanding list of cell types, and containing DNA, RNA, and cytosolic and/or membrane-associated proteins [2]. The notion that such microvesicles may play a role in the false positive contamination of host cells by labeled donor cells has been reported recently in the literature [3].

A variety of literature suggests that human adipose-derived stromal/stem cells (ASCs) are immunotolerant/immunoprivileged and can survive for prolonged periods in immunocompromised and even immunocompetent animals [4–7]. In this study we investigated the survival of human ASCs in both immunocompetent and immunocompromised murine models, delivered either as single-cell suspensions or as self-assembled three-dimensional spheroids. The extent of human cell engraftment was quantified over time using PCR detection of an endogenous retrovirus (ERV-3) present in all human cells, but not present in rodent cells. Since the ERV-3 genetic sequence is present as only a single copy per human cell, its detection is directly proportional to the number of human cells present. Using this sensitive and specific method of human cell detection, we demonstrate that nearly 90% of human ASCs that were viable upon injection are undetectable by day 10 after administration into nude mice. Human cell clearance is even faster in immunocompetent animals.

## Materials and methods

### Isolation and culture expansion of human adipose-derived stromal/stem cells

Human adipose tissue samples were obtained from elective surgical procedures under Institutional Review Board approval at the University of Virginia. We obtained all necessary consent from any patients involved in the study. ASCs were isolated as described previously [8, 9]. Briefly, samples were washed, enzymatically dissociated with Liberase Blendzyme (Roche Applied Science, Indianapolis, USA), and filtered to remove debris [9]. After centrifugation, pelleted cells were recovered and washed. Contaminating erythrocytes were removed by osmotic buffer, and the cells were plated onto tissue culture plastic and culture expanded in adherent monolayer culture in xenogeneic-free growth medium with 1% human serum (LM1%) [10]. After three passages, culture-expanded ASCs were membrane dye (DiI) labeled according to the manufacturer’s protocol (Molecular Probes, Inc., Eugene, OR, USA). Some labeled cells were formed into self-assembling spheroids in hanging drop [11, 12]. Mouse embryonic fibroblast cells (3 T6) were obtained from University of Virginia cell culture core facility and were cultured in Dulbecco’s modified Eagle’s medium/F12 high glucose with 10% fetal bovine serum.

### DiI labeling of adipose-derived stromal/stem cells

ASCs were suspended at a density of 1 × 10^6^/ml in culture medium. Five microliters of DiI cell labeling solution were added to the cell suspension, followed by gentle mixing with a pipette. The mixture was incubated at 37°C for 20 minutes. Next, the labeled suspension tubes were centrifuged at 1,500 rpm/200 × *g* force for 5 minutes at 37°C. This was followed by removal of supernatant and resuspension of the cells in Dulbecco’s modified Eagle’s medium/F12 high glucose with 10% fetal bovine serum at 37°C. This washing procedure was repeated two more times.

### Adipose-derived stromal/stem cell implantation into mice

Procedures were performed with approval of the University of Virginia Animal Care and Use Committee. Two strains of mice were used. Thirty-six immunocompetent wildtype (C57BL/6NCr) mice and 36 immunocompromised (Athymic NCr-nu/nu) mice were anaesthetized using ketamine and randomly treated with 300,000 cells either in suspension or preaggregated into spheroids (10 spheroids each comprised of 30,000 cells), followed by appropriate postoperative pain control. Cells delivered as suspensions were injected subcutaneously and into the inguinal region, while cells formulated as three-dimensional spheroids were delivered through an incision into the inguinal fat pad of mice. Implants composed of nonviable cells/spheroids served as parallel controls, implanted in the contralateral side in a randomized, blinded fashion. Nonviable cell implants were generated by overnight incubation at −80°C, thawing at room temperature and confirmed as nonviable with trypan blue dye exclusion and Cell Proliferation Reagent WST-1 (Roche Applied Science).

### Harvesting and processing of tissues

Three sets of mice (each set comprising 12 immunocompetent mice and 12 immunocompromised mice) were harvested on days 3, 10 and 21 after implantation. Through random sampling, one-half of the mice from each harvest time point were assigned to be used for histology and one-half of the mice for human cell quantification by PCR detection of ERV-3. The histology specimens were fixed in 10% neutral buffered formalin and were embedded in paraffin while the PCR samples were frozen at −80°C.

### Quantification of human adipose-derived stromal/stem cells

Real-time PCR detection of the human/primate-specific ERV-3 was used to evaluate and quantify the presence of human ASCs. Of note, the ERV-3 gene is known to reside at a single locus (on human chromosome 7), enabling a direct correlation between ERV-3 levels and human cell numbers [13]. The primers for the human specific ERV-3 gene were designed as described previously [14, 15]: forward, 5-ATG GGA AGC AAG GGA ACT AAT G; reverse, 5-CCC AGC GAG CAA TAC AGA ATT T (Integrated DNA Technologies, Coralville, Iowa, USA). Preserved samples from injection sites were frozen with liquid nitrogen and ground to powder using a mortar and pestle. DNA extraction was performed with DNAzol (Molecular Research Centre, Cincinnati, Ohio, USA) according to the manufacturer’s protocol. DNA extract from cultured ASCs served as a positive control (that is, ASCs 100%) and DNA extract from an untreated mouse was used as a negative control (that is, ASCs 0%).

Standards were prepared by combining cultured human ASCs and mouse embryonic fibroblast cells (3 T6) in known ratios (ASCs 4.76%, 0.498%, 0.05%, and 0.005%). Accordingly, standards were generated from the mixture of defined numbers (5 × 10^4^, 5 × 10^3^, 5 × 10^2^ and 5 × 10) of human ASCs with 10^6^ mouse embryonic fibroblast cells. Genomic DNA was extracted from these preparations according to experimental protocol. Real-time quantitative PCR with 96-well optical plates was performed and analyzed using an icycler iQ (BioRad, Hercules, CA, USA). Each reaction was performed using 4.5 μl DNA specimen added to 8 μl PCR reagent mixture comprised of SYBR green and forward and reverse primers. Extracted DNA was assessed for quality and quantity using GeneQuant Pro (Amersham Biosciences, Piscataway, NJ, USA) and each sample was run at 1:10 and 1:50 dilutions in duplicate. The PCR conditions used were: first step, 95°C for 15 minutes; and second step, 45 cycles each with 30 seconds at 95°C (denaturation), 30 seconds at 60°C (annealing) and 30 seconds at 72°C (extension). The threshold cycle (C_T_) was defined as the first cycle number in a PCR amplification above baseline and during the exponential increase period, with 40 as the maximum allowable value. Appropriate amplification was determined by melt curve analysis, with an ERV-3 melting temperature of 87.5°C.

**Figure 1:**
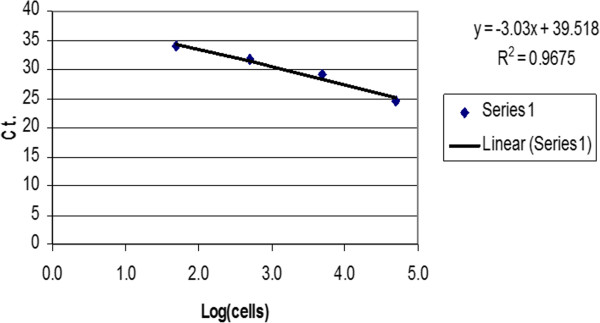
**Standard sample of cells mixture analyzed using real-time PCR.** Ct, cycle threshold.



For generation of a standard curve, six defined mixtures/ratios of human ASCs and mouse embryonic fibroblast cells (3 T6) were analyzed. The amplification curves and C_T_ values obtained are presented in Table 1. Log[human ASCs] linearly correlated with C_T_ values, with a correlation coefficient of 0.97 (Figure 1). From these results, an equation depicting the relationship between C_T_ values and human ASCs was obtained as follows:

**Table 1:** **Real-time PCR results**

Mouse 3 T6 cells	Human ASCs	Human ASC %	C_T_(***n*** = 6)	STD
1,000,000	50,000	4.760	24.6	0.3884
1,000,000	5,000	0.498	29.2	0.2566
1,000,000	500	0.050	31.6	0.3536
1,000,000	50	0.005	33.9	0.0707
1,000,000	0	0	NA	NA
0	50,000	100	24.2	0.2887

Real-time PCR results for single-cell suspension mixtures used to create a standardized curve. ASC, adipose-derived stromal/stem cell; CT, cycle threshold; NA, not available; STD, = Standard Deviation.

### Histology and immunohistochemistry

Tissue sections were stained with hematoxylin and eosin, and representative sections were prepared for immunohistochemical staining by deparaffinization with xylene and rehydration through a graded ethanol series. A heat-mediated antigen retrieval technique that included a 20-minute boil in 0.01 M citrate buffer, pH 6.0 (Fisher Scientific, Waltham, MA, USA) was used. After cooling for 1 hour, two separate 5-minute washes in Tris-buffered saline/Tween20 pH 7.4 (Trizma base; Sigma; Tween20; J.T. Baker, St Louis, MO, USA), followed by two 5-minute washes in phosphate-buffered saline were performed. The sections were incubated in 2% horse serum (Sigma, Austin, TX, USA) prepared in phosphate-buffered saline with 0.5% gelatin from cold water fish skin, to inhibit nonspecific binding of the primary antibody. Following incubation in blocking serum, the sections were incubated in primary antibody rat anti-mouse Mac-2 (Cedarlane, Burlington, ON, Canada), dilution 1:1,000, in a humidified chamber for 1 hour. Following this, two 5-minute washes in 0.5% gelatin from cold water fish skin/phosphate-buffered saline were performed. The sections were then incubated in secondary antibody Alexa Fluor 488 donkey anti-rat (Invitrogen, Carlsbad, CA, USA) for 1 hour in a humidified chamber at room temperature. This was followed by three 5-minute washes in phosphate-buffered saline, prior to aqueous mounting (Fisher Scientific).

The immunostained slides were imaged using a confocal Nikon Eclipse TE 2000-E2 microscope (Nikon, Melville, NY, USA) equipped with a 60× Nikon oil immersion objective

### Statistical analysis

The percentage of ERV-3 was obtained with real-time PCR as the fraction of ERV-3 remaining at the implantation site on days 3, 10 and 21. Data were analyzed using SPSS 19.0 (IBM Corp. Released 2010. IBM SPSS Statistics for Windows, Version 19.0. Armonk, NY, USA). Using an independent-samples *t* test, we compared the percent ERV-3 between the ASCs injected as a cell suspension versus spheroids for different time points (days 3, 10 and 21), separately for the two murine species. We also compared the percent ERV-3 between immunocompetent mice versus immunocompromised mice for the time points studied.

## Results

### Animal survival

All mice survived the surgical procedure of cell implantation. Out of 72 treated animals, 71 survived to experimental endpoint. One athymic NCr-nu/nu mouse injected with spheroids died on day 7.

### PCR for adipose-derived stromal/stem cell presence and quantification

**Figure 2:**
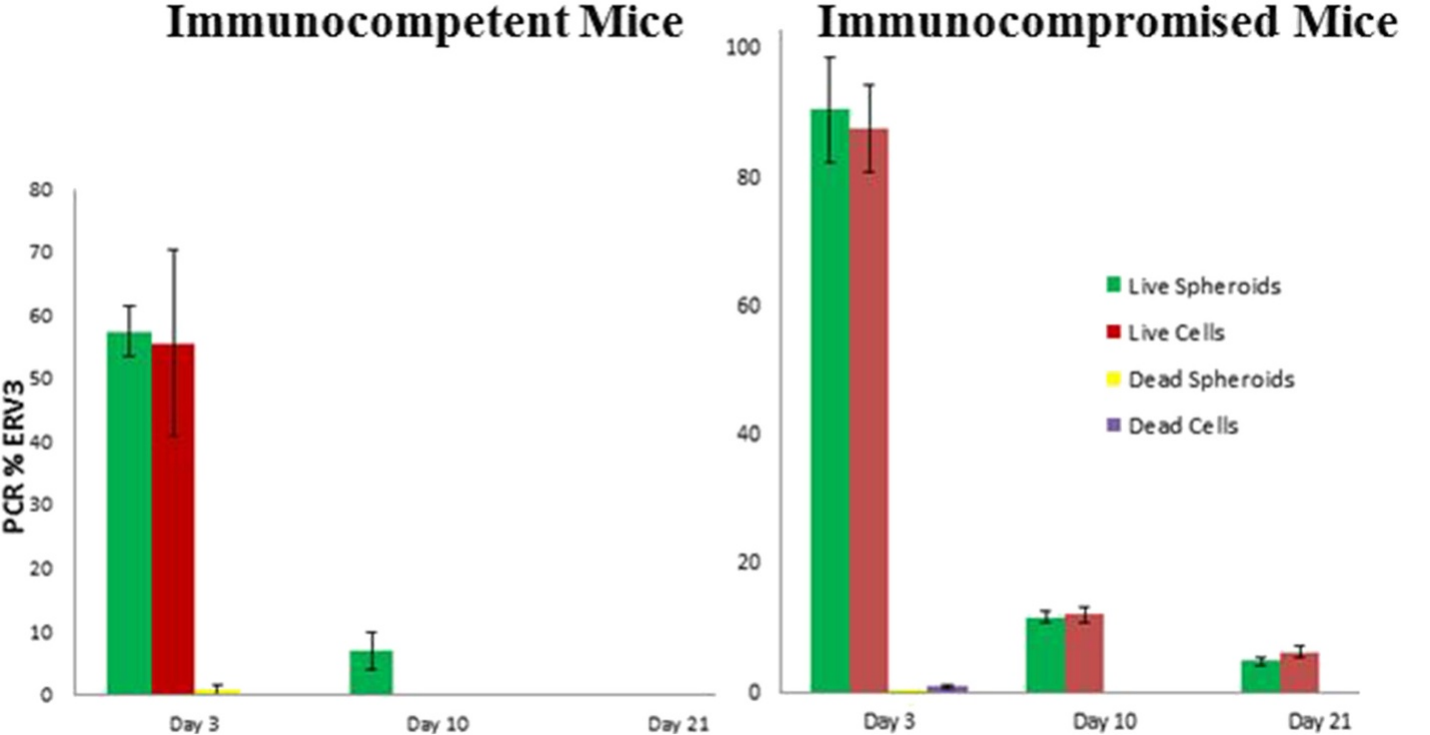
**Graphical representation of adipose-derived stromal/stem cell persistence (% ERV-3) in immunocompetent mice versus immunocompromised mice.** Error bars represent ± 1 standard error of *n* = 3. ERV-3, endogenous retrovirus 3.

Real-time PCR was performed using ERV-3 amplification to assess human cell presence in harvested tissue specimens from injection/implantation sites. The persistence of human ASCs is correlated to the percent ERV-3 amplification [13–15], and our study shows that when viable ASCs are injected *in vivo*, an increased percentage of cells can be detected relative to nonviable ASC implants, regardless of formulation (cell suspension versus spheroids). As expected, the number of detectable ASCs implanted as either cell suspension or spheroids was greater in immunocompromised mice at all time points as compared with immunocompetent mice, with statistically significant differences on days 10 and 21 after implantation (*P* <0.05). However, there were no statistical differences in the PCR detection of ASCs over time when implanted as cell suspension compared with those implanted as three-dimensional spheroids (Table 2, Figure 2).

**Table 2:** **Survival of adipose-derived stromal/stem cells**

Immunocompetent mice					Immunocompromised mice			
% ERV-3	Viable injection		Nonviable injection		Viable injection		Nonviable injection	
	Spheroids	Cells	Spheroids	Cells	Spheroids	Cells	Spheroids	Cells
Day 3 (*n* = 3)	57.5 ± 3.9	55.6 ± 14.7	0.9 ± 0.8	0	90.6 ± 8.2	87.6 ± 6.8	0.1 ± 0.03	0.9 ± 0.3
Day 10 (*n* = 3)	7.1 ± 3.0	0	0	0	11.7 ± 0.8	12.1 ± 1.3	0	0
Day 21 (*n* = 3)	0	0	0	0	4.8 ± 0.6	6.3 ± 0.9	0	0

Percent survival of adipose-derived stromal/stem cells (% ERV-3) measured with real-time PCR. Values represent mean ± standard error of *n* = 3. ERV-3, endogenous retrovirus 3.

### Histology and immunohistochemistry

**Figure 3:**
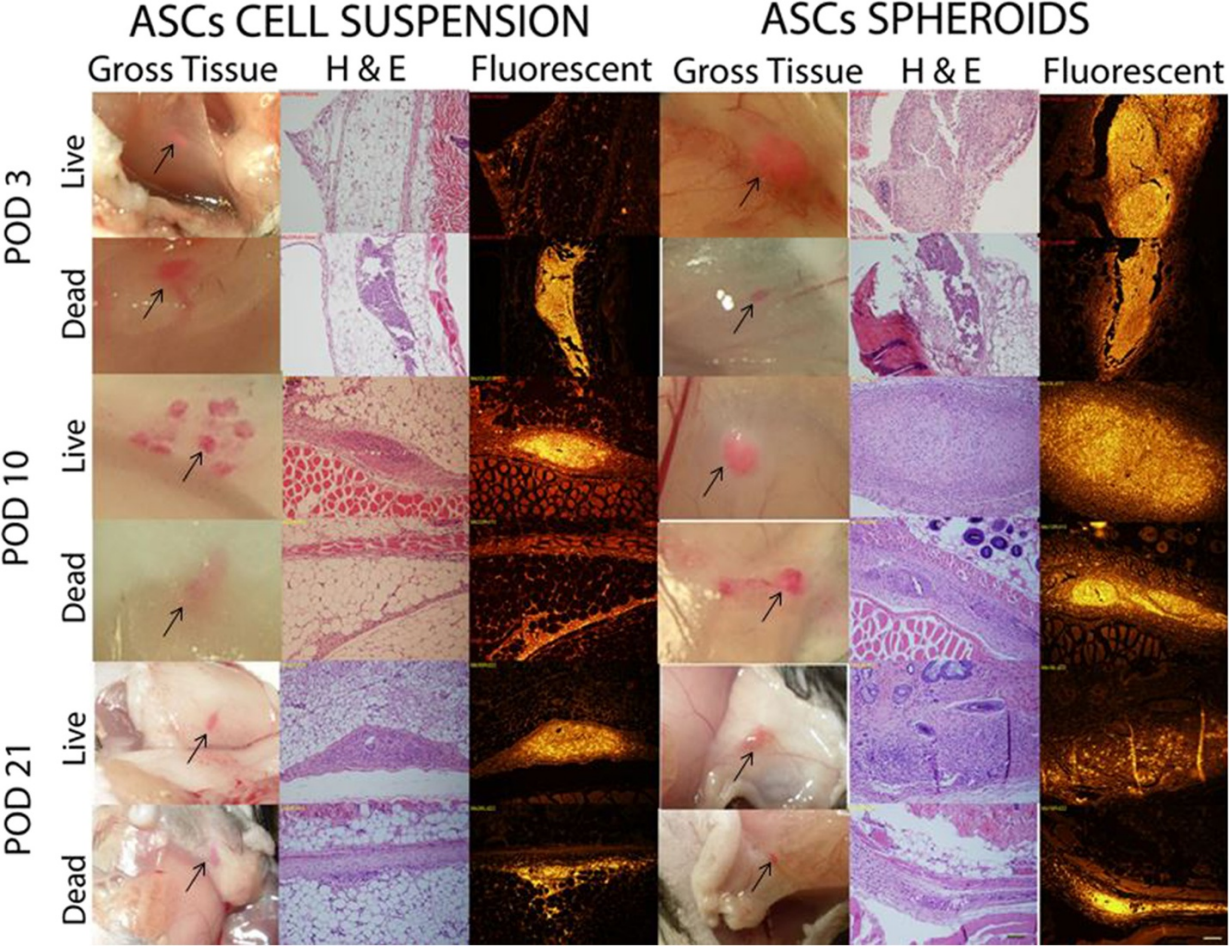
**Immunocompetent mice injected with adipose-derived stromal/stem cells in suspension and in spheroids.** Tissues harvested on days 3, 10 and 21 are represented in different rows. First column, photographs on gross inspection (arrows indicate injection sites); second column, hematoxylin and eosin (H & E)-stained sections; third column, fluorescent images of same sections. Scale bar on lower right corner: 200 μm. ASC, adipose-derived stromal/stem cell.

**Figure 4:**
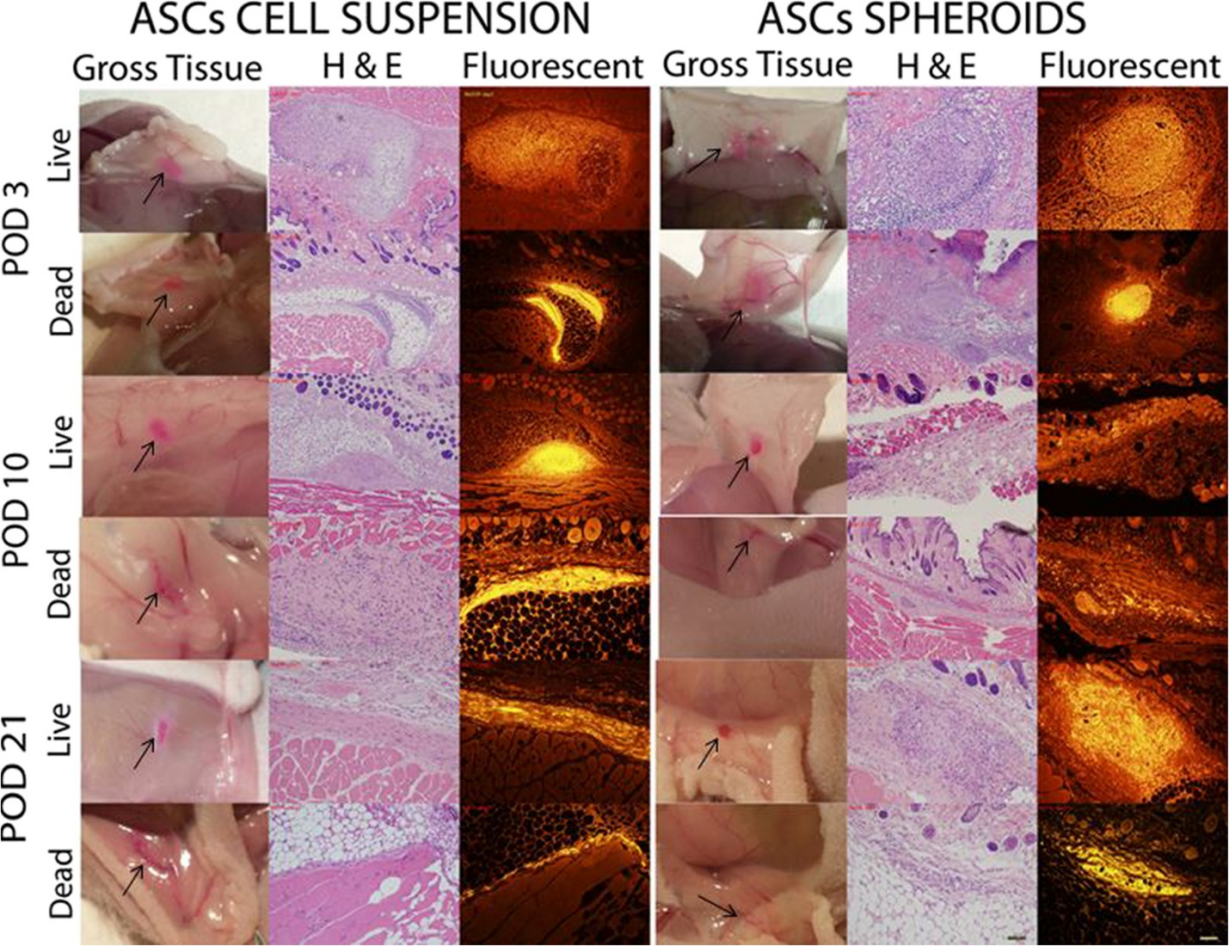
**Immunocompromised mice injected with adipose-derived stromal/stem cells in suspension and in spheroids.** Tissues harvested on days 3, 10 and 21 are represented in different rows. First column, photographs on gross inspection (arrows indicate injection sites); second column, hematoxylin and eosin (H & E)-stained sections; third column, fluorescent images of same sections. Scale bar on lower right corner: 200 μm. ASC, adipose-derived stromal/stem cell.

**Figure 5:**
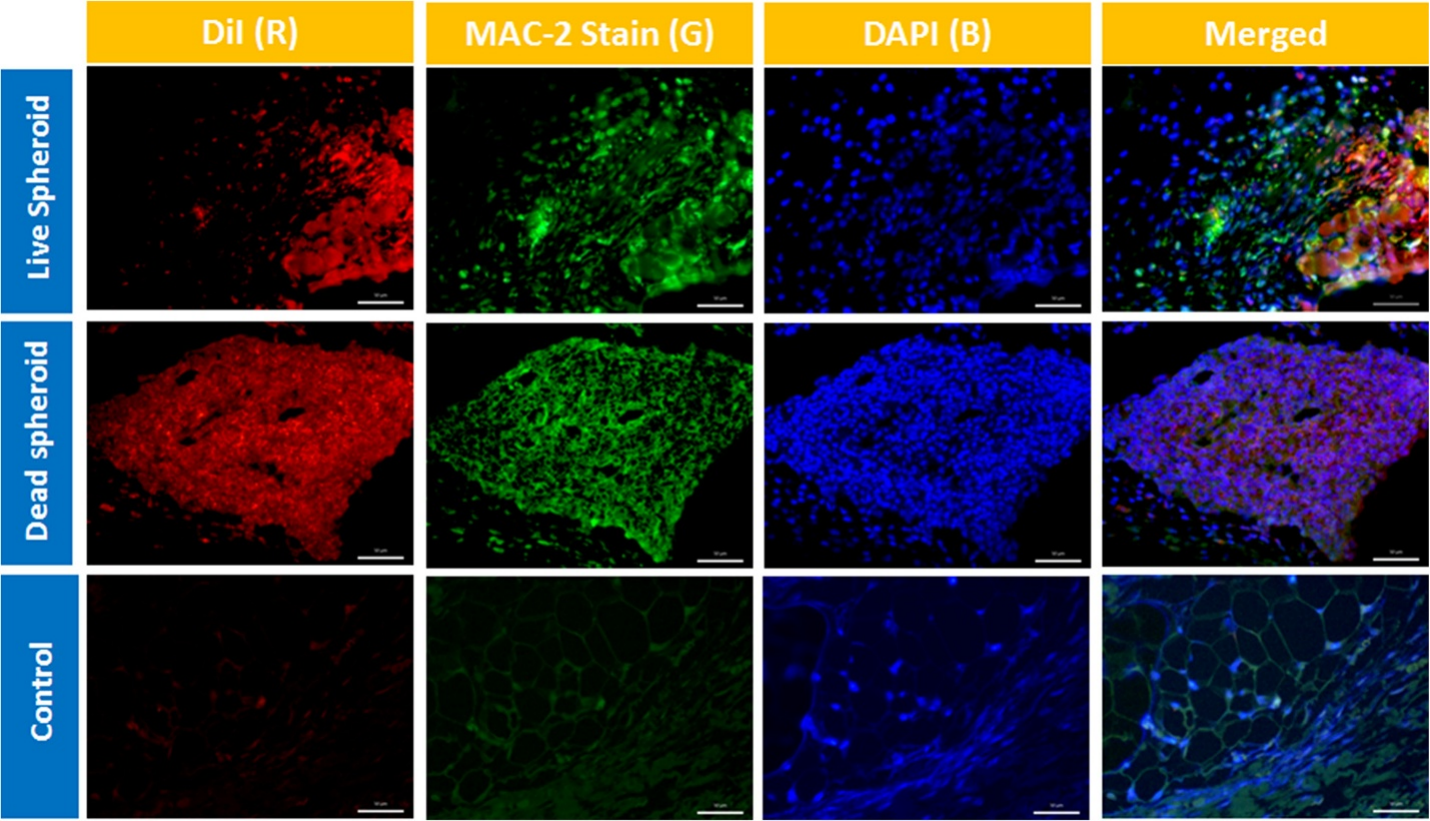
**Immunocompetent mice injected with live and nonviable adipose-derived stromal/stem cell spheroid.** Top row: immunocompetent mice injected with live adipose-derived stromal/stem cell (ASC) spheroids harvested on day 3. First column, images with DiI membrane dye (red); second column, sections with Mac-2 stain (Alexa 488; green); third column, images with 4′,6-diamidino-2-phenylindole (DAPI; blue); fourth column, merged fluorescent images of the same sections. Middle row: Immunocompetent mice injected with live and nonviable adipose-derived stromal/stem cell spheroids and harvested on day 3. Bottom row: non-injected control tissue from immunocompetent mice. Scale bars: 50 μm.

**Figure 6:**
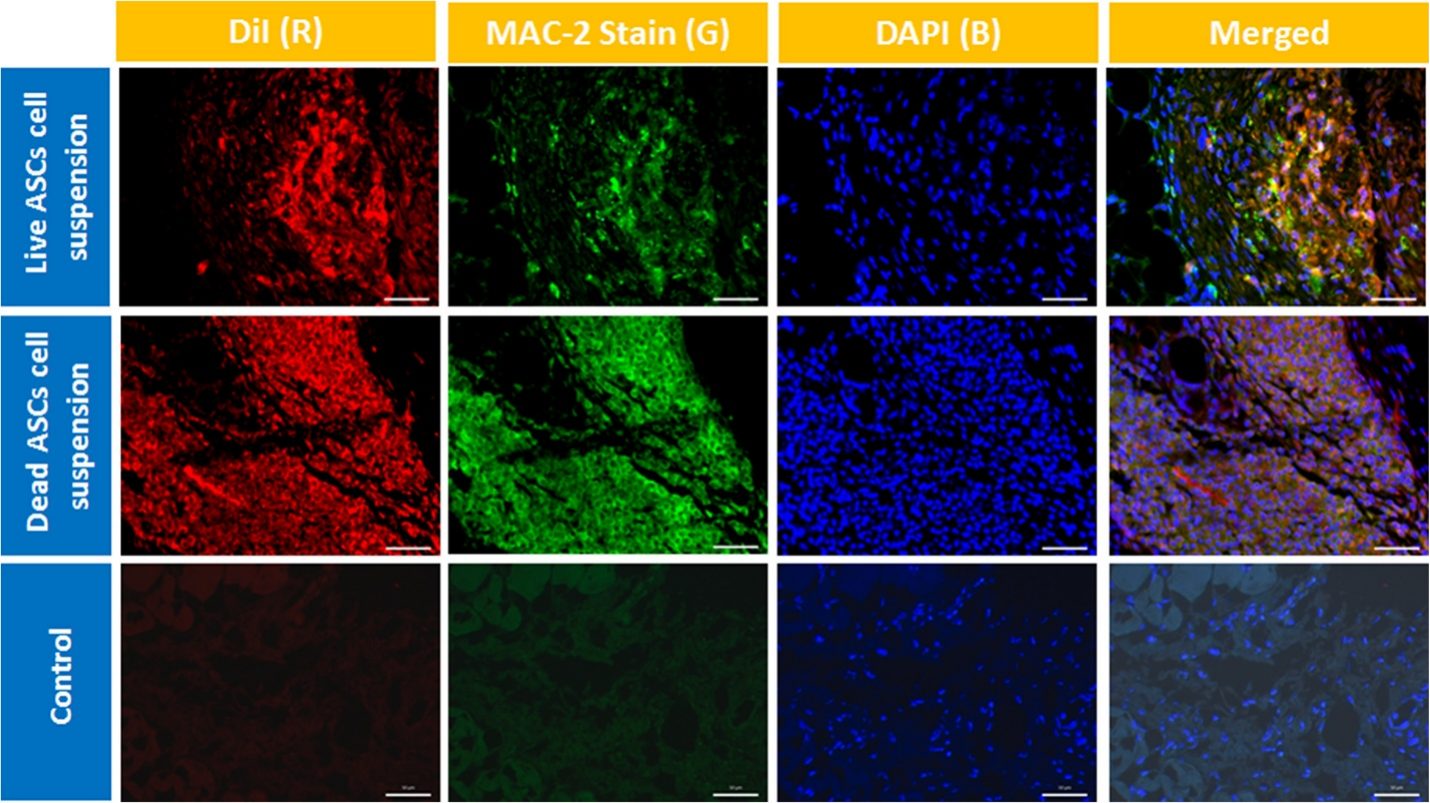
**Immunocompromised mice injected with live and nonviable adipose-derived stromal/stem cell suspensions.** Top row: immunocompromised mice injected with live adipose-derived stromal/stem cell (ASC) cell suspensions harvested on day 3. First column, images with DiI dye (red); second column, sections with Mac-2 stain (Alexa 488; green); third column, images with DAPI 4′,6-diamidino-2-phenylindole (DAPI; blue); fourth column, merged fluorescent images of the same sections. Middle row: nonviable ASC cell suspensions injected into immunocompromised mice and harvested on day 3. Bottom row: non-injected control tissue from immunocompromised mice. Scale bars: 50 μm.

On gross observation, the DiI-labeled implants were readily identifiable as pink blushes at the implantation site throughout the entire study period, although the intensity subjectively waned with later time points. Using fluorescent microscopy, the DiI-labeled human adipose-derived cell implants were readily visible as a fluorescent signal that was localized to the region of implantation for the entire 3-week study period. The fluorescent signal was easily detectable with a high signal-to-noise ratio. The fluorescent signal arising from nonviable cell implants was indistinguishable from that arising from viable cell implants (Figures 3 and 4). Detection of human cells using highly specific PCR-mediated methods showed that <1% of implanted nonviable cells were detectable at the implantation site on day 3 despite the presence of a robust fluorescent signal for up to 3 weeks. On the other hand, up to 90% of implanted viable cells were detectable by PCR on day 3, but this rapidly declined to <5% by day 21, once again despite the presence of a robust fluorescent signal. In all cases, the implanted cells were noted to generate a robust macrophage infiltrate, seen with Mac-2 immunostaining that co-localized with the DiI fluorescent signal (Figures 5 and 6).

**Figure 7:**
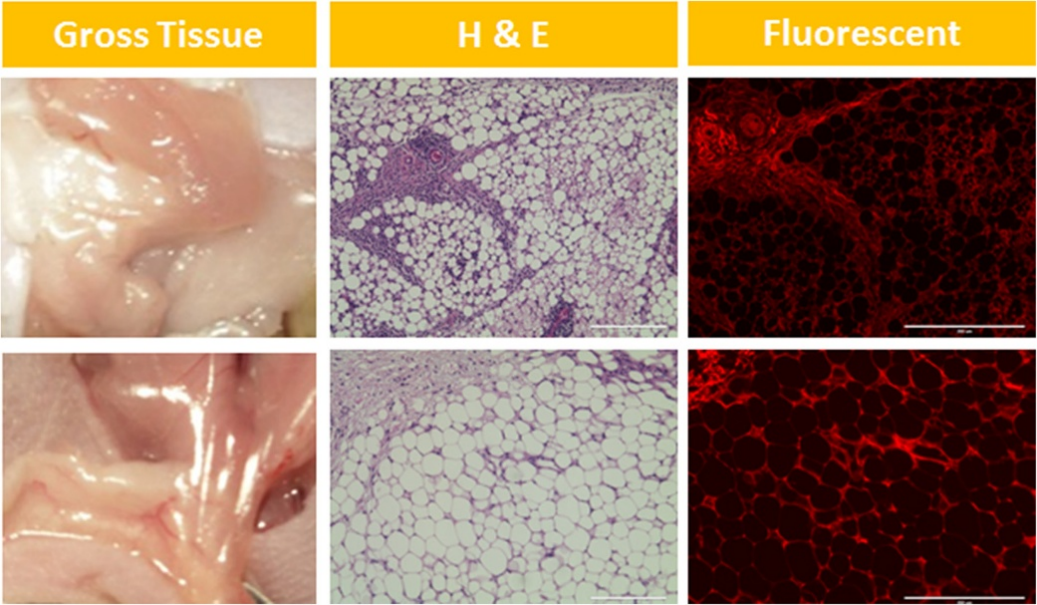
**Non-injected control tissues seen on gross inspection, hematoxylin and eosin stain and fluorescent microscopy.** Top row: immunocompetent mice. Bottom row: immunocompromised mice. H & E, hematoxylin and eosin.

## Discussion

A variety of cell-based therapies stand at the threshold of changing the way medicine is practiced. A growing body of evidence now suggests that cell therapies act primarily via paracrine effects [16, 17]. However, questions remain unanswered as to how many cells are needed and how long such cells are needed at a given site in order to effect a reproducible response. To answer these questions, reliable quantitative methods are required that are both specific and sensitive. In other words, one of several important issues related to the future understanding and translation of such therapies to the clinic pertains to quantifying and optimizing cell survival and biodistribution after cell delivery. This challenge remains critical even for pivotal preclinical studies that are necessary to support the safety and efficacy of emerging therapies.

Given the growing interest in exploiting adipose tissue as an abundant cell source, our objective was to evaluate human ASC persistence after implantation into immunocompetent and immunocompromised mice using fluorescent membrane labeling techniques for histological tracking, and PCR detection of human-specific genetic sequences for confirmatory and quantitative analysis. Both sensitive and specific, real-time PCR for ERV-3 detects as few as 50 human cells in 1,000,000 murine cells (0.005%) [14, 15], compared with detection of 5,000 cells/organ reported in green fluorescent protein studies [18]. ERV-3 is present in the human genome as only one copy per cell, on chromosome 7 [13, 14]. As such, ERV-3 can be used to detect and quantify the presence of human cells, and do so without need for cellular manipulation. However, it does not provide histological or morphological data. Our data show that <1% of nonviable ASCs implanted were detectable by PCR detection of ERV-3, 72 hours after implantation. Although detection of ERV-3 does not give a direct confirmation of cell viability, it is conceivable that a close correlation does exist between cell viability and the ability to detect a specific DNA sequence with PCR. It is known, for example, that large DNA fragmentation occurs as early as 5 minutes after the onset of apoptosis. Further breakdown into smaller fragments continues over 2 to 24 hours [19, 20].

Based on our PCR data, it appears that human ASCs are cleared quite rapidly in the murine strains used in this study, with clearance in immunocompetent mice occurring faster than in immunocompromised mice. More specifically, nearly 90% of cells that were viable at injection are undetectable by day 10, and only 5% are detectable at the implant site at 21 days. It is possible that a majority of the cells migrate away from the injection site by this early time point. However, the robust fluorescent (DiI) signal that is visible at the implantation sites (Figures 3, 4 and 7) argues strongly against this possibility.

Somewhat surprisingly, human cell clearance also appears to be rapid in the immunocompromised Athymic NCr-nu/nu mouse model, raising concern for suitability of this strain for xenogeneic studies. The rapid undetectability of human cells *in vivo* is associated with a robust infiltration of host macrophages – even in the athymic mouse. Although mature T cells are missing in athymic mice, the B cells, dendritic cells and granulocytes are all relatively intact, and there is a compensatory increase in macrophages and natural killer cell activity [21]. The co-localization of Mac-2 staining with fluorescent DiI label/signal suggests strongly that ASC implantation stimulates a rapid and robust macrophage infiltrate which correlates with decreasing (human) cell numbers as measured by ERV-3 quantification (Table 2). Indeed, our results show that a significant amount of DiI signal is located within Mac-2 stained macrophages (Figures 5 and 6). The DiI fluorescent signal remained visible throughout the entirety of our study time period; however, it appears that human ASC-related dye transfer and/or persistence can occur in the context of macrophage-mediated phagocytosis of human cells [22, 23], via the exchange of membrane microdomains [3], and/or via microvesicle and/or exosome [2, 24] transfer. It is possible that the fluorescent DiI stain is itself immunogenic and may accelerate the engulfment of human cells. There could also be tissue-specific and/or species-specific differences in the macrophage response to implanted cells. In this study, we injected human ASCs subcutaneously and into the inguinal fat pad. Adipose tissue is known to contain resident macrophages and can also efficiently recruit additional macrophages when inflammatory stimuli arise [25–27]. It is conceivable that in other tissues the time course or extent of this phenomenon may look different. In addition to tissue-specific responses, it is unclear whether our findings would be similar in other immunocompromised murine models, such as the nonobese diabetic/severe combined immunodeficiency mouse model. Both of these variables deserve further interrogation.

This study also compared the persistence of human ASCs delivered as single-cell suspensions versus three-dimensional spheroids. Contrary to our original hypothesis, this study did not demonstrate any statistical difference in the persistence of ASCs formulated and delivered as three-dimensional spheroids compared with those delivered as cell suspensions. Three-dimensional spheroid cultures are widely believed to better mimic the *in vivo* condition. They exhibit a higher degree of structural complexity and homeostasis, analogous to tissues and organs, including the presence of self-generated extracellular matrix [11]. For these reasons, we believed that cells delivered as spheroids would succumb less to anoikis, and remain more localized and robust than similar cells delivered as a single-cell suspension. However, the ERV-3 data do not support this hypothesis, perhaps because the macrophage infiltrate is similar regardless of the implant formulation.

As summarized in Table 3, a wide variety of cell labeling and tracking methods have been reported in the literature. There is notable discrepancy in the findings of these studies and direct comparison is difficult due to differences in cell source/type, recipient species, cell labeling method and concentration, cell dose, and cell delivery method. For example, Lassailly and colleagues report findings for lipophilic dyes (for example, DiI) similar to ours, but find amine reactive dyes to be biocompatible and associated with little contamination [3]. Weir and colleagues, on the other hand, found that amine reactive dyes displayed rapid signal loss over the course of a week [28]. Vilalta and colleagues reported the persistence of 25% of human ASCs in athymic mice for up to 32 weeks using virally mediated transduction of luciferase [6], and Bai and colleagues reported the persistence of 10% of human ASCs in SCID mice using a similar labeling method [4]. Although no single method or technique of cell tracking has been proven optimal, each method ought to be reconsidered in light of the emerging literature on microvesicles, and membrane fusion events.

**Table 3:** **Previous studies of implanted cell persistence and migration** ***in vivo***

Reference	Species of donor and recipient	Method and site of delivery	Cell labeling method	Findings
[4]	Donor, human ASCs; Recipient, SCID mice	5 × 10^6^cells injected intramyocardially in the peri-infarct region	Transduction with luciferase, GFP	10 weeks: 10% of the human ASCs were localized at the site of injection for 10 weeks. No migration detected. 3.5% differentiated into cardiomyocytes or endothelial cells.
[3]	Donor, human HL60 cells; recipient, NOD/SCID mice	Intravenous injection of 20 × 10^6^ cells stained with DIR. Rest of the cell labeling techniques were studied *in vitro*	Lipophilic dyes: DiI, DiD, DiR, PKH26	2 weeks: lipophilic dyes lead to rapid contamination of neighboring cells. CFSE showed good biocompatibility and staining efficiency and showed little contamination. DDAO was toxic to cells. Quantum dots provided heterogeneous staining that is not suitable for intravital microscopy (IVM). IRDye 800CW had suboptimal excitation by the 633 nm lasers used for IVM in this study
			Amine reactive dye: CFSE, DDAO-SE	
			Nano crystals: quantum dots 70S	
			Antibodies: IRDye 800CW	
[30]	Donor, porcine ASCs; recipient, Pigs	Subcutaneous implantation of cells seeded in collagen scaffold	BrdU labeling	4 weeks: BrdU-labeled ASCs were present but no quantification was done
[31]	Donor, MCF7 human breast cancer cells, human cord blood-derived cells, human NeoHep cells, human hepatopancreatic precursors; recipient, NOD/SCID mice	Injection into left lobe of liver, 7.5 × 10^5^ human NeoHep or cord blood cells; tail vein injection, 5 × 10^6^ MCF7 cells; intrapancreatic injection, 5 × 10^5^ hepatopancreatic precursor cells; intracardiac transplantation, 5 × 10^5^ hepatopancreatic precursor cells	DiI and red fluorescent nanoparticles Qdot655	3 weeks: FISH for human-specific Alu sequence and mouse major satellite showed that though many of DiI-labeled cells were human in origin, some were phagocytosed by murine cells. Qdot655 faded during the FISH procedure.
[6]	Donor, human ASCs; recipient, BALB/C nu/nu mice	5 × 10^6^cells injected i.m. or i.v.	Transduction with luciferase	75% of cells were lost in first week, the remainder were stable for up to 32 weeks
[28]	Donor, sheep MSCs; recipient, Merino-cross sheep	Intramuscular injection	DiI labeling and CFSE	DiI-labeled MSCs showed dye retention for 6 weeks. CFSE showed rapid signal loss over 8 days
[32]	Donor, human ASCs; recipient, BALB/C nu/nu mice	10^6^ cells injected i.m., s.c., i.v., i.p. or enclosed in a fibrin matrix	Lipofection and electroporation with luciferase, GFP	3 weeks: cells migrated and accumulated at the ventral side. A higher fibrinogen concentration limited cell mobility in the fibrin matrix

ASC, adipose-derived stromal/stem cell; CFSE, carboxyfluorescein succinimidyl ester; FISH, fluorescent in situ hybridization; GFP, green fluorescent protein; i.m., intramuscularly; i.p., intraperitoneally; i.v., intravenously; MSC, mesenchymal stem cell; NOD/SCID, nonobese diabetic/severe combined immunodeficiency; s.c., subcutaneously.

Microvesicles are produced by most cells, including mesenchymal stem cells and ASCs [1], and they can carry and transmit RNA, DNA, cytosolic proteins, and cell membrane proteins. Moreover, it is known that environmental stressors can stimulate the release of microvesicles from cells [29]. As such, cytosolic and membrane-bound proteins such as luciferase and green fluorescent protein can potentially be transmitted to host cells via fusion events and/or microvesicle secretion, resulting in false positive microenvironmental contamination. Similarly, detection of human cell surface labels such as human leukocyte antigen or of human-specific genetic sequences such as Alu can also be a result of microvesicle-mediated false positive contamination of the microenvironment. Although it remains supposition at this point in time, it is statistically plausible that the more prevalent a given protein or genetic sequence is within a cell, the higher the likelihood it will be transferred to a neighboring cell/microenvironment via a microvesicle or similar mechanism. By this logic, Alu elements – which are the most abundant transposable elements in the human genome – as well as cytosolic proteins such as green fluorescent protein and luciferase, are more likely to be transferred to neighboring cells than is the single DNA copy per cell of ERV-3. This assumption certainly requires further studies to confirm or repute, but is worth consideration when interpreting pertinent studies. A comparison of various cell tracking methods, including their relative advantages and disadvantages, is presented in Table 4.

**Table 4:** **Comparison of contemporary cell tracking methods**

Tracking method – mechanism	Disadvantages	Advantages
GFP, luciferase – DNA transfer can be mediated virally (transduction), via liposomes (lipofection), or by electrical parameters (electroporation/transfection)	- Genetic manipulation of cells may alter their function	- Human ASCs with GFP or luciferase resume proliferation normally
	- Since they are cytosolic in location; the vector (mRNA/DNA) and/or the protein (GFP) could be transmitted to host cells via fusion elements and/or microvesicle secretion, resulting in contamination. Hence, there is concern for false positivity and their detection may not equate to viability of donor cells	- Detection is sensitive to *in vivo* non-invasive bioluminescence imaging
	- Technique requires serial passages, not suitable for use with fresh uncultured cells	
	- Many mammalian tissues have endogenous fluorescence	
BrdU – this nuclear marker is a thymidine analog that replaces (3H) thymidine and can penetrate cell membranes to incorporate into newly synthesized DNA strands of actively proliferating cells	- Optimal labeling requires longer incubation time	- BrdU labeling has no effects on the ASC differentiation/proliferation and is not cytotoxic
	- Not suitable for non-invasive methods of detection	
	- Does not indicate viability	
	- Cells lose BrdU rapidly with serial passages	
Lipophilic dyes (DiI, DiR) –long-chain carbocyanine dyes with long aliphatic tails that incorporate into the lipid regions of the cell membranes	- Rapidly contaminates neighboring cells by macrophage-mediated phagocytosis, exchange of membrane microdomains, microvesicle and/or exosome transfer	- Easy technique for labeling and identification of cells
	- May be cytotoxic to cells	
	- The dye fades with serial passages	
Amine reactive probes (CFSE) – these diffuse into cells and react with cytosolic amine-containing residues to form dye–protein adducts that are retained	- The dye is toxic to ASCs	- Good staining efficiency
	- Not suitable for *in vivo* non-invasive imaging	
	- Does not correlate with viability	
	- Contamination of neighboring cells can occur via macrophage-mediated phagocytosis, microvesicle and/or exosome transfer	
Nanoparticles – small crystals made up of inorganic molecules; for example, iron oxide, cadmium	- Can be toxic to cells in high concentration and detection is difficult with low concentrations	- Photostable, remain resistant for long periods of time
	- Contamination of neighboring cells can occur via phagocytosis, microvesicles and/or exosome transfer	- Can be used for *in vivo* non-invasive imaging
	- Does not correlate with viability	
Real-time PCR for endogenous retroviral sequence (ERV-3) – the gene is present as a single copy in the human genome and so can be used to detect the presence of transplanted human cells in animal models	- Not suitable for non-invasive methods of detection	- Gives a quantitative estimate of number of cells
	- False positives can occur by macrophage-mediated phagocytosis but this is very low	- Very sensitive and specific
		- The gene is already present in the human cells, so there is no need to stain the cells
FISH detection of human-specific cell surface markers or Alu sequences	- Alu sequences occur in large numbers in the primate genome, which makes higher likelihood of a false positive detection by transmission to host cells/macrophages via microvesicles/exosomes	- The gene is already present in the human cells, so there is no need to stain the cells
	- Not suitable for non-invasive methods of detection	
	- Does not correlate with viability	

ASC, adipose-derived stromal/stem cell; BrdU, 5-Bromo-2-deoxyurudine; CFSE, carboxyfluorescein succinimidyl ester; ERV-3, endogenous retrovirus 3; FISH, fluorescent in situ hybridization; GFP, green fluorescent protein.

## Conclusion

In summary, these studies demonstrate that when human ASCs are implanted into the subcutaneous and inguinal fat tissue of mice, almost all cells are undetectable within 3 weeks using sensitive and specific molecular techniques. In contrast, a robust fluorescent signal from commonly used membrane dyes is readily detectable by microscopy – even from nonviable cell implants. The co-localization of DiI signal with the MAC-2 stain revealed that DiI is taken up by macrophages by phagocytosis and perhaps other mechanisms of cell transfer. Although clearance of ASCs occurred faster in immunocompetent mice compared with immunocompromised mice, there were no significant differences in the persistence of ASCs delivered as cell suspensions versus those implanted as spheroids. In short, with the emerging evidence of microvesicle formation by ASCs and other modes of host cell contamination, we propose that ERV-3 detection by PCR is a useful method for detecting and quantifying the presence of human cells in xenogeneic models. Further studies are needed to help delineate the advantages and limitations of the various methods available for cell labeling, tracking, identification, and quantification.

## Abbreviations

ASC: adipose-derived stromal/stem cell
C_T_: threshold cycle
DiI: membrane dye
ERV-3: endogenous retrovirus 3.
